# Allergen-specific immunotherapy in rhinitis patients is associated with milder COVID-19 symptoms and improved quality of life

**DOI:** 10.1016/j.clinsp.2026.100998

**Published:** 2026-05-24

**Authors:** Hanqiao Li, Renkang Wang, Benquan Yu, Jun He, Yibo Hu, Zuozhong Xie, Zi'an Xiao

**Affiliations:** a Department of Otolaryngology Head and Neck Surgery, The Second Xiangya Hospital, Central South University, Changsha, Hunan Province, China; b Institute of Otology, Central South University, Changsha, Hunan Province, China; c Laboratory of Otolaryngology Head and Neck Cancer, The Second Xiangya Hospital, Central South University, Changsha, Hunan Province, China; d Department of Otolaryngology Head and Neck Surgery, Stanford University School of Medicine, Stanford, CA, USA; e Clinical Research Center, The Second Xiangya Hospital, Central South University, Changsha, Hunan, China

**Keywords:** Allergic rhinitis, Allergen-specific immunotherapy, COVID-19, Clinical characteristics, Prognosis

## Abstract

**Purpose:**

This retrospective study investigated the impact of Allergic Rhinitis (AR) patients receiving Allergen-specific Immunotherapy (AIT) on Coronavirus Disease 2019 (COVID-19) infection, clinical characteristics, and prognosis.

**Methods:**

This was a single-center, retrospective cohort study that included patients with AR who received subcutaneous desensitization with Aloege or sublingual desensitization from 10 December to 31 March 2023 at Changsha The Second Xiangya Hospital, China.

**Results:**

The investigation comprised 761 outpatients, including 336 individuals with allergic rhinitis who underwent AIT and 425 healthy subjects. Patients with AR who received AIT treatment showed a significantly lower risk of COVID-19 infection in the multivariate analysis (OR=0.48, *p* = 0.005). The probability of developing hyposmia after contracting COVID-19 was reduced in the experimental cohort compared to the control cohort (OR = 0.39, 95 % CI 0.27‒0.57, *p* < 0.001). Additionally, the test group experienced a reduction in sore throat (OR = 0.32, 95 % CI 0.22‒0.47, *p* < 0.001) and a shorter duration of systemic symptoms (OR = 0.49, 95 % CI 0.34‒0.7, *p* < 0.001) following COVID-19 infection. Furthermore, it was discovered that the impact on quality of life after COVID-19 infection was significantly different between the two groups, with the test group exhibiting a higher quality of life compared to the control group (*p* < 0.001).

**Conclusion:**

During a concentrated or sudden outbreak of COVID-19, patients with AR who received AIT had a lower proportion of hyposmia than the healthy population, and the severity of sore throat and its impact on quality of life were relatively mild.

## Highlights

• AIT linked to lower hyposmia and sore throat risk after COVID-19.
• AIT patients had shorter systemic symptoms and better QoL with COVID.
• First study of AIT's protective effect on COVID-19 using China's post-lockdown cohort.
• AIT immunomodulation may offer broader protection against respiratory viruses.

## Introduction

Allergic Rhinitis (AR) is a common chronic inflammatory condition of the upper respiratory tract with a worldwide prevalence of approximately 10%‒40%.^1^ Atopic individuals experience chronic inflammatory symptoms of the nasal mucosa, such as episodes of sneezing, a runny nose, and nasal congestion, mainly after exposure to allergens. This disease usually affects the patient's daily work and study. The indirect costs of decreased work productivity and attendance are estimated to be 3.2 to 13.5 times greater than the direct medical costs and account for 76% to 93% of the total cost of AR.^2^ There is no cure for AR, and symptoms can be controlled by minimizing allergen exposure and medication to improve quality of life. Allergen-specific Immunotherapy (AIT) is currently the only etiologic treatment capable of modifying disease progression.^3^

Since December 2019, Coronavirus Disease 2019 (COVID-19) has been spreading globally, and its infections usually cause significant upper respiratory tract reactions and systemic symptoms, and even severe pneumonia, respiratory distress, and systemic organ dysfunction leading to death, which has a huge impact on the health of the global population.^4^^,^^5^ Since the beginning of the COVID-19 pandemic until July 2023, the World Health Organization (WHO) has reported 767 million cases of COVID-19, resulting in approximately 6.95 million deaths due to COVID-19 infection, with a case-fatality rate of approximately 1%.^6^ With the intensive study of COVID-19, scientists have found that patients with AR have a lower risk of COVID-19 infection than non-AR patients, and AR has a protective role during COVID-19 infections.^7^, ^8^, ^9^ However, whether the incidence and symptoms of COVID-19 infection in patients with AR receiving AIT are consistent with and impacted by AR has not been studied to date. Since December 7, 2022, when the studied country adjusted the COVID-19 epidemic prevention policy, the country entered the phase of full liberalization of COVID-19, which facilitated the study of this topic. This study aimed to observe the effects on the rate of COVID-19 infection, clinical symptoms, and prognosis in patients with AR who received AIT after the full liberalization of the COVID-19 epidemic prevention policy in China.

## Materials and methods

### Materials

A total of 336 patients with AR who received Arog subcutaneous desensitization therapy or sublingual desensitization therapy in the Department of Otorhinolaryngology-Head and Neck Surgery, the Second Xiangya Hospital, Central South University, were selected as the experimental group from December 10 to March 31, 2023. The control group consisted of 425 healthy individuals without AR, asthma, and other diseases. Those who had been infected with COVID-19 before December 2022 were also excluded.

### Methods

This observational study was reported following the Strengthening the Reporting of Observational Studies in Epidemiology (STROBE) Statement. In this study, a questionnaire survey was used, which included the respondents' gender, age, time of developing AR, AIT method and the duration of AIT, other treatment modalities, the presence of other nasal diseases, whether or not they had been injected with the COVID-19 vaccine, whether or not they had been infected with COVID-19, changes in their sense of smell before and after the infection, time of the emergence of the hyposmia, the recovery of their sense of smell, their systemic symptoms (e.g., fever, headache, sore throat, and generalized muscular aches and pains), the post-infectious bed rest, the proportion of people who had contracted pneumonia following the infection, and the degree to which their quality of life had been affected (including their sleep, moods, and their work and schooling). Written informed consent was obtained from all patients. Ethical approvals for the study were given by The Second Xiangya Hospital of Central South University.

### Statistical analysis

This study used SPSS 26.0 statistical software for statistical data processing. For the measurement data, the Shapiro-Wilk test revealed that it did not conform to a normal distribution, so the median (interquartile range: minimum-maximum) was used to express it, and the rank-sum test was used to make comparisons between groups. The authors used the χ^2^ test for the analysis of count data, and *p* < 0.05 was considered a statistically significant difference. For the analysis of all outcome variables, the authors performed logistic regression analyses using R 4.3.0 statistical software, taking into account multivariate variables such as age, gender, vaccination n-vaccination status, and pre-infection olfactory condition. Model fitting excluded multicollinearity through the variance inflation factor (VIF <2.0) and verified calibration through the Hosmer-Lemeshow test (*p* > 0.05) to ensure the stability and interpretability of the model. The authors considered *p* < 0.05 to be statistically significant.

## Result

### Basic characteristics of the patients with AR in this study

The duration of AR was mainly 6 (4, 10) years 35.71% of the patients with AR also suffered from other nasal diseases such as sinusitis, nasal polyps, deviated septum, and adenoid hypertrophy. All patients with AR in the test group denied a history of nasal tumors. In addition, 4.46% of the patients had asthma, and 27.68% had allergic conjunctivitis. There were also 5.36% of patients with both diseases. 90.18% of patients were treated with subcutaneous injections and 9.82% with Sublingual administration. In addition to AIT, some patients received a combination of nasal spray medication (32.14%) or injections of omalizumab (4.46%). Of these, 28.57% were treated with nasal spray hormones only, 0.89% with nasal spray antihistamines, and 2.68% with nasal spray hormones and antihistamines. In this study, the duration of AIT in patients with AR was concentrated in < 1-year (33.93 %) and 1‒2 years (47.32 %) (see Table 1). According to Appendix Table S3, patients with AR who received AIT with synchronized omalizumab injections had a lower risk of COVID-19 infection (OR=0.16, 95 % CI 0.05‒0.46, *p* < 0.001).

**Table 1:** Basic clinical characteristics of the test group population.

Basic characteristics [(Example) %]	Value
Duration of AR (years)	6 (4, 10)
Associated with other nasal diseases	
Yes	120 (35.71 %)
No	216 (64.29 %)
Associated Diseases	
Asthma	15 (4.46 %)
Allergic conjunctivitis	93 (27.68 %)
Both	18 (5.36 %)
None	210 (62.50 %)
AIT method	
Subcutaneous injections	303 (90.18 %)
Sublingual administration	33 (9.82 %)
Synchronous treatment	
nasal spray medication	108 (32.14 %)
injections of omalizumab	15 (4.46 %)
None	213 (63.39 %)
Types of nasal spray drugs	
Hormones	96 (28.57 %)
Antihistamines	3 (0.89 %)
Both	9 (2.68 %)
None	228 (67.86 %)
The duration of AIT	
Within 1-year	114 (33.93 %)
1‒2 years	159 (47.32 %)
2‒3 years	27 (8.04 %)
>3-years	36 (10.71 %)

AR, Allergic Rhinitis; AIT, Allergen-specific Immunotherapy.

Other nasal diseases referred to as sinusitis, nasal polyps, deviated nasal septum, or adenoid hypertrophy, and all patients with allergic rhinitis in the test group denied a history of nasal tumors. Nasal spray hormone refers to nasal spray of budesonide, mometasone furoate, fluticasone and other corticosteroids.

## Comparison of baseline characteristics and factors associated with COVID-19 infection: univariate and multivariate logistic regression analyses

### Basic characteristics of the two groups of the population

According to the data in Table 2, the two groups had a significant difference in the age distribution (*p* < 0.001). The age of the test group was mainly concentrated at 17 (13, 31.75) years, while the age of the control group was mainly 24 (24, 25) years. There was a significant difference between the two groups in the proportion of shots of the COVID-19 vaccine (*p* < 0.001). The experimental group was mainly vaccinated with 2 (48.21%) and 3 (40.18%) doses of vaccine, while the control group was mainly vaccinated with 3 (67.06%) doses of vaccine. The number of COVID-19 infections in the test group was 300 with a prevalence rate of 89.29%, and the number of COVID-19 infections in the control group was 360 with a prevalence rate of 84.71 %, a statistically non-significant difference (*p* = 0.064). In addition, the differences between the two groups were not statistically significant in terms of gender ratio and whether they were vaccinated against COVID-19 (*p*>0.05). According to Appendix Table S2, vaccinated patients with AR developed hyposmia later after COVID-19 infection compared to unvaccinated patients (OR=16.55, 95 % CI 4.84‒56.64, *p* < 0.001).

**Table 2:** Comparison of the basic characteristics of the two groups.

Characteristic	Control (*n* = 425)	Test (*n* = 336)	Value	p
Gender				
Male	210	189	χ^2^ = 3.518	0.061
Female	215	147		
Age (years)	24 (24, 25)	17 (13, 31.75)	*Z*=−8.108	<0.001
Vaccinated or not				
Yes	405	324	χ^2^ = 0.600	0.439
No	20	12		
Number of vaccination doses				
One	5	9	χ^2^ = 60.92	<0.001
Two	105	162		
Three	285	135		
Four	10	18		
COVID-19 Infection			χ^2^ = 3.419	0.064
Yes	360	300		
No	65	36		

Control, Control group; Test, Test group.

### Logistic regression analysis of factors associated with COVID-19 infection rates: univariate and multivariate results

**Fig. 1:**
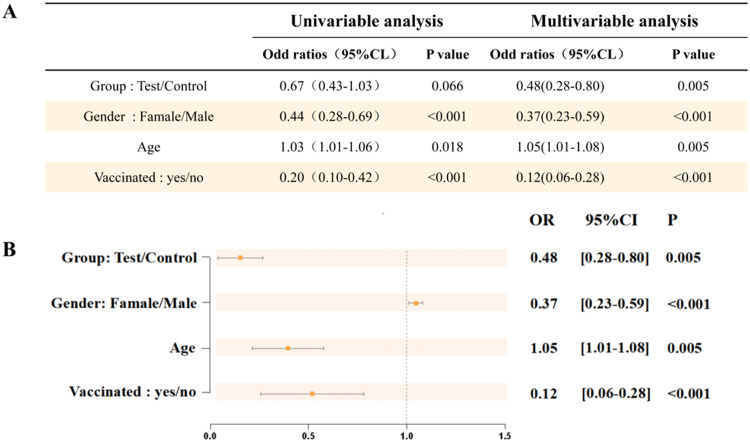
Logistic regression analysis of the infection rate of the novel coronavirus. (A) Univariate and multivariate analysis tables. (B) Multivariate analysis OR forest plot, with the x-axis showing OR values and the y-axis showing population, gender, age, and whether vaccinated.

As presented in Fig. 1, the authors evaluated the impact of different variables on the COVID-19 infection rate through logistic regression analysis. The results of the univariate analysis indicated that gender (females compared to males), vaccination status (vaccinated individuals compared to unvaccinated individuals), and age were all significantly associated with the infection rate (*p* < 0.05). Specifically, the infection risk for females was significantly lower than that for males (OR = 0.44, *p* < 0.001), and the infection risk for vaccinated individuals was significantly lower than that for unvaccinated individuals (OR = 0.20, *p* < 0.001). Moreover, for each additional year of age, the infection risk showed a significant upward trend (OR = 1.03, *p* = 0.018). The multivariate analysis further adjusted for potential confounding factors and revealed that gender (females compared to males, OR = 0.37, *p* < 0.001) and vaccination status (vaccinated individuals compared to unvaccinated individuals, OR = 0.12, *p* < 0.001) still had significant protective effects on the COVID-19 infection rate, and the positive association between age and infection risk became more pronounced (OR = 1.05, *p* = 0.005). Notably, the experimental group showed a significantly lower infection risk compared to the control group in the multivariate analysis (OR = 0.48, *p* = 0.005). In conclusion, gender, vaccination status, and age are all independent predictors of the COVID-19 infection rate.

### Olfactory characteristics and comparison between patients treated with AIT for AR and normal subjects before and after infection with COVID-19

#### Olfactory characteristics of the two groups before and after infection with COVID-19

According to the results in Table 3, in the analysis of the sense of smell before and after infection with COVID-19, there was a significant difference between the two groups of people in terms of the effect on the sense of smell after infection with COVID-19. Specifically, the population in the AR group showed a lower percentage of decreased and significant decrease in the sense of smell after infection with COVID-19 than the control group (*p* < 0.05). However, no significant differences were observed between the two groups in terms of the sense of smell before infection with COVID-19, the time of onset of hyposmia after infection with COVID-19, and the time of recovery of the sense of smell (*p* > 0.05).

**Table 3:** Olfactory characteristics of the two study groups before and after infection with COVID-19.

Characteristic	Control (*n* = 360)	Test (*n* = 300)	Value	p
Pre-infection olfactory function				
Normal	330	267	χ^2^=1.348	0.246
Diminished	30	33		
Olfactory function after infection				
Normal	185	204	χ^2^=18.66	<0.001
Diminished	175	96		
Degree of olfactory dysfunction				
Slightly decreased	85	60	χ^2^=4.835	0.028
Significantly decreased	90	36		
Time of olfactory dysfunction				
Within the first week	145	72	χ^2^=2.339	0.121
Above the first week	30	24		
Olfactory rehabilitation				
Within the first week	90	60	χ^2^=3.075	0.080
Above the first week	85	36		

Control, Control group; Test, test group.

Time of olfactory dysfunction refers to the time of appearance of hyposmia after the COVID-19.

#### Analysis and comparison of olfactory characteristics of the two groups before and after infection with COVID-19

**Fig. 2:**
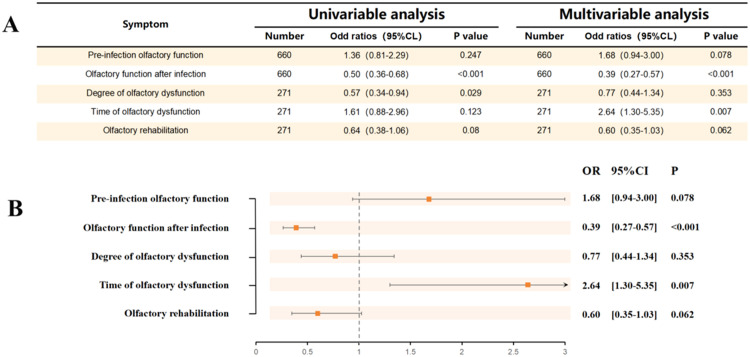
Olfactory characteristics of the two groups. (A) Univariable and multivariable analyses of olfactory characteristics of two groups of study subjects before and after infection with COVID-19. (B) Comparison of olfactory characteristics between two groups of study subjects before and after infection with COVID-19. The x-axis shows the OR value and the y-axis shows olfactory characteristics after infection with COVID-19.

As shown in Fig. 2, patients with AR undergoing AIT exhibited a lower risk of hyposmia after infection with COVID-19 (OR=0.5, 95 % CI 0.36‒0.68, *p* < 0.001). A multifactorial logistic regression analysis, which took into account gender, age, whether or not the vaccine was administered, and pre-infection olfactory status, found that the protective effect persisted (OR = 0.39, 95 % CI 0.27‒0.57, *p* < 0.001). Patients with AR undergoing AIT were also protected against the degree of olfactory decompensation after infection with COVID-19 (OR = 0.57, 95 % CI 0.34‒0.94, *p* < 0.05). After multifactorial logistic regression adjusting for sex, age, whether or not vaccinated, and pre-infection sense of smell, this protective effect did not cease to exist (OR = 0.77, 95 % CI 0.44‒1.34 *p* > 0.05). However, patients with AR undergoing AIT did not differ significantly in the time to onset of hyposmia compared to controls (OR = 1.61, 95 % CI 0.88‒2.96, *p* = 0.123), but the time to onset of hyposmia after adjusting for sex, age, whether or not they had been vaccinated, and pre-infection sniffing would have been relatively late (OR = 2.64, 95 % CI 1.3‒5.35, *p* = 0.007). Table S1, Table S2, and Figures S1 to S4 (see Appendix) report the comparisons between the test and control groups after adjusting for different covariates.

### Characterization and comparison of systemic symptoms and quality of life between patients treated with AIT for AR and normal subjects after infection with COVID-19

#### Characterization of systemic symptoms and quality of life after infection with COVID-19

After infection with COVID-19, there was a significant difference in the degree of sore throat among systemic symptoms between the two groups (*p* < 0.001), with the degree of sore throat being less severe in the AR group. In addition, the two groups also showed statistically significant differences in the number of days of bed rest and the duration of systemic symptoms after infection with COVID-19 (*p* < 0.05), with patients in the AR group having a relatively shorter duration of systemic symptoms and the number of days of bed rest (for details, see Table 4). The impact on quality of life after infection with COVID-19 also showed significant differences between the two groups (*p* < 0.001), with patients with AR having a higher quality of life (see Table 5 for details). However, no statistically significant differences were observed between the two groups in terms of severity of systemic symptoms after infection, bed rest time, degree of fever, incidence of pneumonia, and quality of life during the recovery period after infection with COVID-19 (*p* > 0.05, Tables 4 and 5).

**Table 4:** Prevalence of systemic symptoms after infection with COVID-19 in two study groups.

Characteristic	Control (*n* = 360)	Test (*n* = 300)	Value	p
Degree of systemic symptoms after infection				
Severe	310	252	χ^2^=0.577	0.448
Mild or asymptomatic	50	48		
Bed rest after infection				
Rest within 2-days	215	207		
>2-days' rest	145	93	χ^2^=6.109	0.013
Fever after infection (degrees Celsius)				
Above 38	275	231		
Normal or <38	85	69	χ^2^=0.034	0.853
Sore throat after infection				
sharp pain	155	66		
No pain or mild pain	205	234	χ^2^=32.57	<0.001
Pneumonia after infection				
Yes	35	24		
No	325	276	χ^2^ = 0.596	0.440
Duration of symptoms after infection				
Within 3-days	215	222		
>3-days	145	78	χ^2^ = 14.91	<0.001

Control, Control group; Test, test group.

Rest within 2-days means that bed rest is not required or the number of days of bed rest is within 2-days after infection with COVID-19.

Duration of systemic symptoms after infection indicates the duration of systemic discomforts such as fever, headache, and generalized pain after infection with COVID-19, in addition to cough.

**Table 5:** Extent of impact on quality of life after COVID-19 infection in both groups.

Period	Control	Test	Value	p
Infection period	6 (4, 8)	4 (2, 7.75)	*Z*=−4.662	<0.001
Recovery period	4 (2, 6)	4 (2, 6)	*Z*=−1.432	0.152

Control, Control group; Test, Test group.

No effect on sleep, mood, work, and study after infection with COVID-19 is 0 grade. A higher score means that the symptoms have a greater impact on all aspects of life and the range of values is 0‒10 grades.

#### Analysis and comparison of the characteristics of systemic symptoms after infection with COVID-19 in two groups

**Fig. 3:**
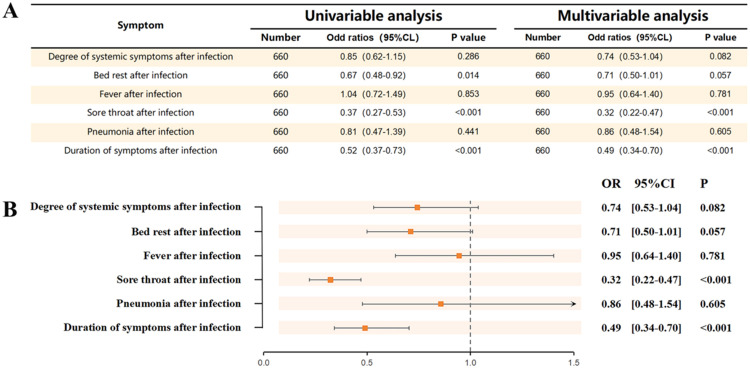
Characterization of systemic symptoms in the two groups. (A) Univariable and multivariable analysis of systemic symptoms after infection with COVID-19 in two groups. (B) Comparison of the characteristics of systemic symptoms after infection with COVID-19 in two groups of subjects. The x-axis shows the OR-value and the y-axis shows the characteristics of systemic symptoms after infection with COVID-19.

According to the results in Fig. 3, the AR group had a shorter duration of bed rest after infection with COVID-19 relative to the control group (OR = 0.67, 95 % CI 0.48‒0.92, *p* = 0.014). However, by performing a multifactorial logistic regression and adjusting for the effects of gender, age, whether or not they were vaccinated, and their sense of smell before infection, the effect of this difference became non-significant (OR = 0.71, 95 % CI 0.5‒1.01, *p* = 0.057).

In addition, the AR group had a protective effect on sore throat after infection with COVID-19 (OR=0.37, 95 % CI 0.27‒0.53, *p* < 0.001). In addition, a protective effect was demonstrated against systemic symptom duration (OR = 0.52, 95 % CI 0.37‒0.73, *p* < 0.001). In a multifactorial logistic regression, the authors adjusted for the effects of gender, age, whether or not the vaccine was administered, and pre-infection sense of smell. The results showed that this protective effect remained significant. For the duration of systemic symptoms, the adjusted OR was 0.49 (95 % CI 0.34‒0.7, *p* < 0.001), and for sore throat, the adjusted OR was 0.32 (95 % CI 0.22‒0.47, *p* < 0.001). Detailed comparative results are shown in Tables S1 and S2 and Figures S1 through S4 in the Appendix.

## Discussion

AR is a chronic inflammatory disease of the nasal mucosa, and its atopic symptoms often affect patients' quality of life. A survey of living conditions in patients with AR showed that 66 % of adults and 43 % of children reported sleep disturbances among 100 moderate-severe patients, and patients with moderate-severe AR showed more signs of anxiety, depression, fatigue, social impairment, and cognitive dysfunction than patients with milder forms of the disease.^10^ Symptoms of AR often affect work productivity, and studies have found that AR reduces work productivity in 36 %‒59 % of patients, with 20 % of these patients reporting decreased work attendance.^11^ Indirect costs of decreased work productivity and attendance were estimated to be 3.2 to 13.5 times higher than direct medical costs and accounted for 76 % to 93 % of total AR costs.^12^ The indirect costs of reduced productivity and attendance due to AR are higher than those of diabetes, migraine, anxiety, or asthma.^13^

There is no cure for AR, and symptoms can be controlled by minimizing allergen exposure and medication to improve quality of life. AIT is currently the only etiologic treatment capable of modifying the progression of the disease^14^ with two routes of administration, subcutaneous and sublingual, and a total course of AIT of three years, with patients continuing to benefit after discontinuation of medication after a full course of therapy, with effects lasting for at least three years.^14^

The results of this study indicate that age, gender, and vaccination status all influence the COVID-19 infection rate among the population. Additionally, patients with AR who received AIT treatment demonstrated a significantly lower risk of COVID-19 infection in the multivariate analysis (OR=0.48, *p* = 0.005). This finding is consistent with the study by Wang et al.,^15^ which investigated 1246 patients with allergic rhinitis who received immunotherapy and found that the COVID-19 infection rate among these patients was lower than that of the general population, similar to the present results. These observations suggest that AIT may offer a mild protective effect against COVID-19 infection. However, Emel et al.^16^ observed 419 patients with AR and found no statistically significant differences in infection rate, pneumonia, or hospitalization rate between the AIT group and the control group. This discrepancy could be related to differences in sample size and confounding factors.

However, in the present study, observations suggest that AIT might have a protective effect on patients with AR in terms of symptoms and quality of life after infection with COVID-19. It has been proposed that receiving AIT reduces the severity of symptoms of COVID-19 infection, which appears more pronounced in patients with comorbid asthma.^17^ This protective effect was further verified in a large-sample cohort study by Zang et al.^18^ involving 1116 subjects, the risk of symptom occurrence after COVID-19 infection was significantly reduced in AR patients who received immunotherapy.

The relationship between AIT for AR and COVID-19 infections needs to be further investigated. The authors hypothesize that the observed association might be explained by the immunomodulatory mechanisms of AIT. AIT for AR involves inducing differentiated regulatory T- and B-cells (Tregs, Bregs) to produce cytokines such as IL-10 and TGF-β by down-regulating type 2 helper T-cells (Th2) while up-regulating type 1 helper T-cells (Th1). These changes could potentially reduce the inflammatory response and type II cytokine production and induce the production of IgE blockade to achieve therapeutic effects.^19^ Larenas-Linnemann et al.^20^ hypothesized that AIT might restore the normal immune function against COVID-19 infection by modulating the function of immune-responsive cells in the body, thereby enhancing the normal immune function against COVID-19 infection. It is possible that simultaneously inducing an increase in Tregs, Bregs and anti-inflammatory cytokines may help to control the excessive inflammatory response induced by COVID-19. This mechanism could potentially be related to the reduction of symptoms after COVID-19 infection in the allergic patients who underwent AIT in this study.

Regarding the lower COVID-19 infection rates in AR patients compared to non-AR subjects, this might be related to a significant reduction in cellular expression of Angiotensin-Converting Enzyme 2 (ACE2) receptor in the upper airways of patients with AR, as reported in the literature.^21^ Such a reduction could potentially reduce viral binding to cellular receptors during COVID-19 infection, thus possibly reducing the infection rate. Additionally, the lower admission and mortality rates in eosinophilic COVID-19-infected patients may be associated with reduced bronchial epithelial-ACE2 expression in patients with high allergic sensitization.^22^ The mechanism behind increased ACE2 expression in patients with low blood eosinophilia remains unspecified.^22^

Inhaled corticosteroid therapy has been shown to inhibit coronavirus replication.^23^ Similarly, intranasal corticosteroid application appears to reduce the risk of hospitalization, serious illness, and death in patients with COVID-19 infection,^24^ and the related mechanism of action is not clear. However, in Appendix Table 3, the use of nasal spray corticosteroids in patients with AR was not observed to reduce the rate of COVID-19 infection in this study.

This study has several limitations that warrant consideration. First, the cross-sectional design and primary reliance on unvalidated, retrospective questionnaires introduce a substantial risk of recall and reporting bias, which may affect the accuracy of the self-reported outcomes. Second, although the authors controlled for major confounding factors through multivariate adjustment, the potential for residual confounding remains, as unmeasured or unknown factors could have influenced the results. Third, the sample size, particularly for analyzing differences in COVID-19 infection rates, was relatively limited, resulting in insufficient statistical power; consequently, the finding of a reduced infection risk associated with AIT must be interpreted as preliminary and requires validation in larger cohorts. Finally, the generalizability of these conclusions are constrained by the demographic composition of the study population, which consisted predominantly of adolescents and young adults, thereby limiting its applicability to middle-aged and elderly populations.

In conclusion, the present research indicates that AR patients who received AIT treatment demonstrated a significantly lower risk of COVID-19 infection in the multivariate analysis. Compared with the healthy population, they experienced a lower proportion of hyposmia and a relative reduction in the degree of sore throat, the duration of systemic symptoms, and the impact on quality of life compared with the healthy population. Despite the limitations of this study, this study provides a preliminary exploration of the association between AIT and COVID-19 infection in patients with AR, and these findings provide evidence as to whether patients with AR should be immunized to prevent or mitigate COVID-19 damage.

## Ethics approval

This study was approved by the human study ethics committees at The Second Xiangya Hospital of Central South University. The approval number is LYF20220261. All patients provided written informed consent for participation in the study. It was conducted in accordance with the principles of the Declaration of Helsinki.

## Clinical trial number

Not applicable.

## Data availability

The datasets used and/or analyzed during the current study are not publicly available due to institutional policy but are available from the corresponding author on reasonable request.

## Authors' contributions

All authors made a significant contribution to the work reported, whether that is in the conception, study design, execution, acquisition of data, analysis and interpretation, or in all these areas; took part in drafting, revising or critically reviewing the article; gave final approval of the version to be published; have agreed on the journal to which the article has been submitted; and agree to be accountable for all aspects of the work.

## Funding

This study was supported by the 10.13039/501100004735Natural Science Foundation of Hunan Province (Grant Nos. 2025JJ50712 and 2023JJ20087) and the Natural Science Foundation of Changsha (Grant No. kq2208327).

## Declaration of competing interest

The authors declare no conflicts of interest.

## Appendix Supplementary materials

Image, application 1

## Footnotes

Supplementary material associated with this article can be found, in the online version, at doi:10.1016/j.clinsp.2026.100998.
