# Supplementary material for: Allergen-specific immunotherapy in rhinitis patients is associated with milder COVID-19 symptoms and improved quality of life
Source: Clinics (Sao Paulo). 2026 May 24;81:100998. doi: 10.1016/j.clinsp.2026.100998 (PMC13224361; doi:10.1016/j.clinsp.2026.100998)
Supplement: Supplementary file 1 [file mmc1.docx]

CLINICS-D-24-01190_Supplementary Material

***Table S1. Univariate analysis was conducted to examine the clinical symptoms in patients following infection with COVID-19.***

|  |  | Group | | Sex | | Age | | **Vaccine** | | **Pre-infection olfactory function** | |
| --- | --- | --- | --- | --- | --- | --- | --- | --- | --- | --- | --- |
|  |  | Control | AR | Male | Female | 1-24 | >24 | No | Yes | No | Yes |
| Pre-infection olfactory function | RR (95%CI) | Reference | 1.36（0.81-2.29） | Reference | 0.33（0.19-0.59） | Reference | 1.28（0.75-2.19） | Reference | 176081249.4 | Reference |  |
|  | p-value |  | 0.247 |  | <0.001 |  | 0.37 |  | 0.998 |  |  |
| Olfactory function after infection | RR (95%CI) | Reference | 0.5（0.36-0.68） | Reference | 1.46（1.07-2） | Reference | 1.31（0.95-1.79） | Reference | 0.96(0.38-2.41) | Reference | 36.59(11.34-118.08) |
|  | p-value |  | <0.001 |  | 0.017 |  | 0.098 |  | 0.925 |  | <0.001 |
| Degree of olfactory dysfunction | RR (95%CI) | Reference | 0.57（0.34-0.94） | Reference | 1.7（1.05-2.77） | Reference | 1.76（1.07-2.91） | Reference | 0.51(0.12-2.18) | Reference | 0.12(0.06-0.27) |
|  | p-value |  | 0.029 |  | 0.032 |  | 0.026 |  | 0.365 |  | <0.001 |
| Time of olfactory dysfunction | RR (95%CI) | Reference | 1.61（0.88-2.96） | Reference | 2.73（1.4-5.3） | Reference | 14.53（4.4-47.98） | Reference | 0.14(0.3-0.59) | Reference | 0.88(0.42-1.83) |
|  | p-value |  | 0.123 |  | 0.003 |  | <0.001 |  | 0.008 |  | 0.726 |
| Olfactory rehabilitation | RR (95%CI) | Reference | 0.64（0.38-1.06） | Reference | 1.32（0.81-2.14） | Reference | 0.73（0.45-1.19） | Reference | 1376567902 | Reference | 0.78(0.44-1.4) |
|  | p-value |  | 0.08 |  | 0.26 |  | 0.208 |  | 0.999 |  | 0.412 |
| Degree of systemic symptoms after infection | RR (95%CI) | Reference | 0.85（0.62-1.15） | Reference | 0.91（0.67-1.23） | Reference | 0.8（0.59-1.1） | Reference | 0.5(0.19-1.32) | Reference | 0.81(0.48-1.37) |
|  | p-value |  | 0.286 |  | 0.531 |  | 0.168 |  | 0.161 |  | 0.437 |
| Bed rest after infection | RR (95%CI) | Reference | 0.67（0.48-0.92） | Reference | 1.68（1.22-2.32） | Reference | 1.45（1.04-2.01） | Reference | 1.6(0.57-4.5) | Reference | 1.7 (1.01-2.87) |
|  | p-value |  | 0.014 |  | 0.002 |  | 0.026 |  | 0.373 |  | 0.046 |
| Fever after infection | RR (95%CI) | Reference | 1.04（0.72-1.49） | Reference | 0.88（0.61-1.26） | Reference | 0..72（0.49-1.04) | Reference | 1.54(0.57-4.12) | Reference | 0.45(0.26-0.78) |
|  | p-value |  | 0.853 |  | 0.488 |  | 0.08 |  | 0.392 |  | 0.004 |
| Sore throat after infection | RR (95%CI) | Reference | 0.37（0.27-0.53） | Reference | 1.39（1.01-1.93） | Reference | 1.07（0.77-1.49） | Reference | 1.42(0.51-4) | Reference | 0.99(0.57-1.72) |
|  | p-value |  | <0.001 |  | 0.045 |  | 0.686 |  | 0.504 |  | 0.979 |
| Pneumonia after infection | RR (95%CI) | Reference | 0.81（0.47-1.39） | Reference | 2.67（1.49-4.79） | Reference | 1.23(0.71-2.13) | Reference | 163768186 | Reference | 1.08(0.45-2.62) |
|  | p-value |  | 0.441 |  | 0.001 |  | 0.46 |  | 0.998 |  | 0.864 |
| Duration of symptoms after infection | RR (95%CI) | Reference | 0.52（0.37-0.73） | Reference | 0.98（0.71-1.35） | Reference | 1.1(0.79-1.52) | Reference | 1.44(0.51-4.06) | Reference | 0.9(0.52-1.58) |
|  | p-value |  | <0.001 |  | 0.89 |  | 0.587 |  | 0.487 |  | 0.719 |

***Table S2. Analysis of clinical symptoms in patients following infection with COVID-19 using a multivariate approach.***

|  |  | **Group** | | **Sex** | | **Age** | | **Vaccine** | | **Pre-infection olfactory function** | |
| --- | --- | --- | --- | --- | --- | --- | --- | --- | --- | --- | --- |
|  |  | Control | AR | Male | Female | 1-24 | >24 | No | Yes | No | Yes |
| Pre-infection olfactory function | **RR (95%CI)** | Reference | 1.68（0.94-3） | Reference | 0.33（0.18-0.59） | Reference | 1.92（1.05-3.52） | Reference | 105808585.6 | Reference |  |
|  | **p-value** |  | 0.078 |  | <0.001 |  | 0.035 |  | 0.998 |  |  |
| Olfactory function after infection | **RR (95%CI)** | Reference | 0.39（0.27-0.57） | Reference | 2（1.4-2.85） | Reference | 0.81（0.56-1.19） | Reference | 0.93（0.36-2.45） | Reference | 55.21（16.76-181.91） |
|  | **p-value** |  | <0.001 |  | <0.001 |  | 0.29 |  | 0.885 |  | <0.001 |
| Degree of olfactory dysfunction | **RR (95%CI)** | Reference | 0.77（0.44-1.34） | Reference | 0.95（0.54-1.67） | Reference | 2.01（1.16-3.5） | Reference | 0.97（0.22-4.32） | Reference | 0.11（0.05-0.26） |
|  | **p-value** |  | 0.353 |  | 0.864 |  | 0.014 |  | 0.97 |  | <0.001 |
| Time of olfactory dysfunction | **RR (95%CI)** | Reference | 2.64（1.3-5.35） | Reference | 2.39（1.11-5.16） | Reference | 16.55（4.84-56.64） | Reference | 16.55（4.84-56.64） | Reference | 1.02（0.42-2.45） |
|  | **p-value** |  | 0.007 |  | 0.027 |  | <0.001 |  | <0.001 |  | 0.968 |
| Olfactory rehabilitation | **RR (95%CI)** | Reference | 0.6（0.35-1.03） | Reference | 1.47（0.87-2.5） | Reference | 0.7（0.41-1.17） | Reference | 1439312434 | Reference | 0.96（0.51-1.82） |
|  | **p-value** |  | 0.062 |  | 0.151 |  | 0.174 |  | 0.999 |  | 0.902 |
| Degree of systemic symptoms after infection | **RR (95%CI)** | Reference | 0.74（0.53-1.04） | Reference | 0.88（0.64-1.21） | Reference | 0.72（0.51-1.01） | Reference | 0.43（0.16-1.16） | Reference | 0.84（0.5-1.43） |
|  | **p-value** |  | 0.082 |  | 0.43 |  | 0.056 |  | 0.096 |  | 0.525 |
| Bed rest after infection | **RR (95%CI)** | Reference | 0.71（0.5-1.01） | Reference | 1.81（1.29-2.53） | Reference | 1.19（0.83-1.7） | Reference | 2.08（0.73-5.96） | Reference | 2.01（1.17-3.45） |
|  | **p-value** |  | 0.057 |  | <0.001 |  | 0.347 |  | 0.174 |  | 0.012 |
| Fever after infection | **RR (95%CI)** | Reference | 0.95（0.64-1.4） | Reference | 0.86（0.59-1.26） | Reference | 0.74（0.49-1.11） | Reference | 1.45（0.53-3.97） | Reference | 0.44（0.25-0.76） |
|  | **p-value** |  | 0.781 |  | 0.432 |  | 0.146 |  | 0.47 |  | 0.004 |
| Sore throat after infection | **RR (95%CI)** | Reference | 0.32（0.22-0.47） | Reference | 1.46（1.03-2.06） | Reference | 0.67（0.46-0.97） | Reference | 1.51（0.51-4.41） | Reference | 1.22（0.68-2.17） |
|  | **p-value** |  | <0.001 |  | 0.032 |  | 0.034 |  | 0.455 |  | 0.505 |
| Pneumonia after infection | **RR (95%CI)** | Reference | 0.86（0.48-1.54） | Reference | 2.91（1.6-5.31） | Reference | 1.05（0.58-1.9） | Reference | 246780978.4 | Reference | 1.4（0.56-3.5） |
|  | **p-value** |  | 0.605 |  | <0.001 |  | 0.877 |  | 0.998 |  | 0.476 |
| Duration of symptoms after infection | **RR (95%CI)** | Reference | 0.49（0.34-0.7） | Reference | 0.97（0.69-1.36） | Reference | 0.85（0.59-1.22） | Reference | 1.36（0.47-3.93） | Reference | 0.94（0.53-1.68） |
|  | **p-value** |  | <0.001 |  | 0.858 |  | 0.378 |  | 0.571 |  | 0.844 |

Models adjusted for age, gender, allergic rhinitis, whether or not vaccinated, and pre-infection olfactory function; OR, Odds Ratio; CI, confidence intervals.

***Table S3. Analysis of the impact of asthma and allergic conjunctivitis on the risk of COVID-19 infection in patients with allergic rhinitis. The infection rate of COVID-19 among participants who used certain medications: 90-L 90-L、Antihistamines、Omalizumab and allergen-specific immunotherapy (AIT).***

|  | **Subgroup** | **Variable** | **OR (95%CI)** | **p-value** |
| --- | --- | --- | --- | --- |
| **COVID-19 infection** | **Asthma** | NO | Reference |  |
|  |  | YES | 1.22(0.35-4.23) | 0.751 |
|  | **Allergic conjunctivitis** | NO | Reference |  |
|  |  | YES | 1.55(0.7-3.41) | 0.281 |
|  | **intranasal corticosteroids** | NO | Reference |  |
|  |  | YES | 1.41（0.64-3.12） | 0.394 |
|  | **Antihistamines** | NO | Reference |  |
|  |  | YES | 201934355.4 | 0.999 |
|  | **Omalizumab** | NO | Reference |  |
|  |  | YES | 0.16(0.05-0.46) | <0.001 |
|  | **allergen-specific immunotherapy (AIT)** | **subcutaneous immunotherapy (SCIT)** | Reference |  |
|  |  | **sublingual immunotherapy (SLIT)** | 217816394.4 | 0.999 |


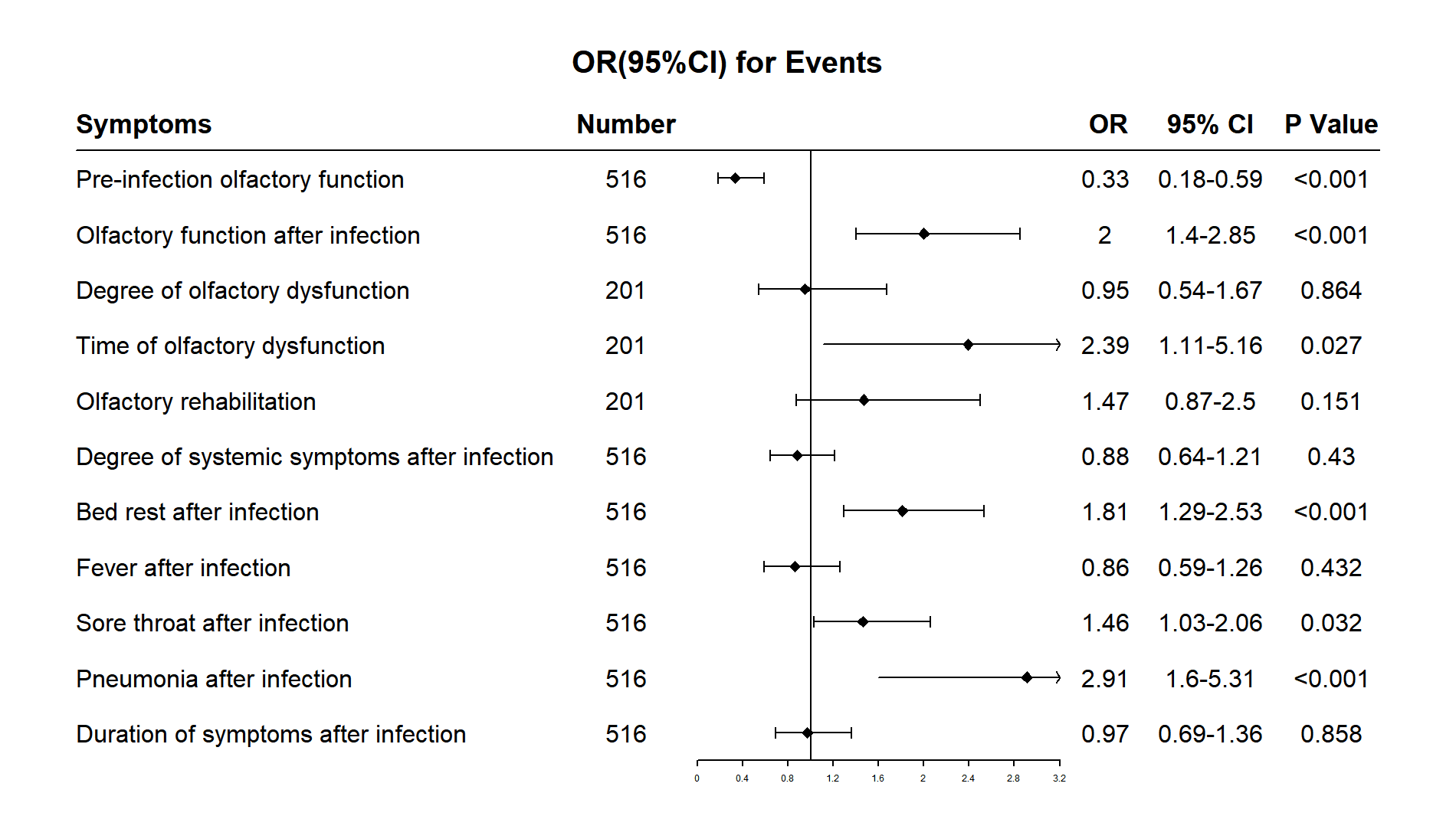


***Figure S1. Forest plot of gender and clinical symptom profile before and after infection with COVID-19***. Models adjusted for age, gender, allergic rhinitis, whether or not vaccinated, and pre-infection olfactory function; OR, Odds Ratio; CI, confidence intervals


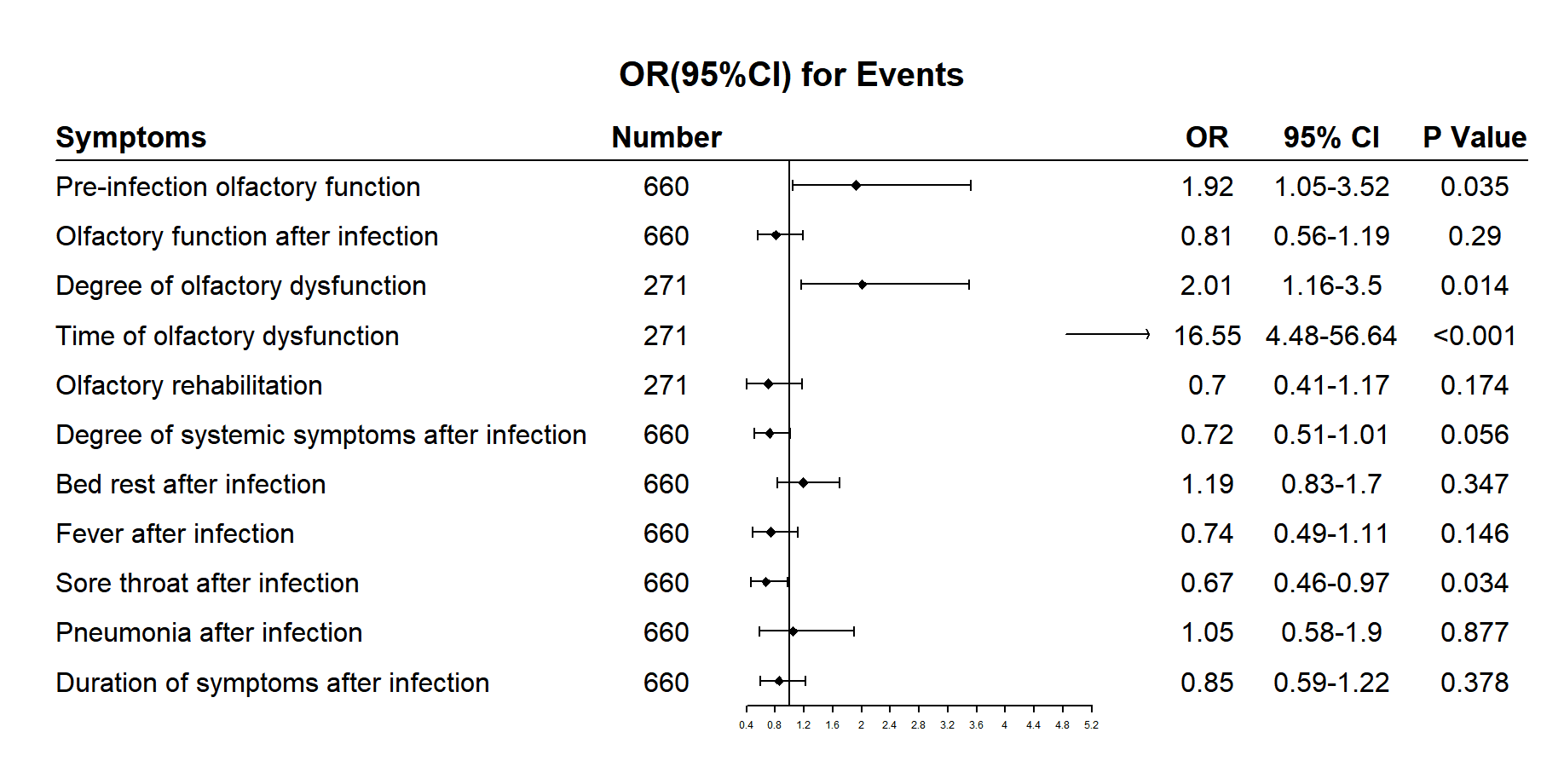


***Figure S2. Forest plot of clinical symptoms and age profile before and after COVID-19 infection.*** Models adjusted for age, gender, allergic rhinitis, whether or not vaccinated, and pre-infection olfactory function; OR, Odds Ratio; CI, confidence intervals.

***
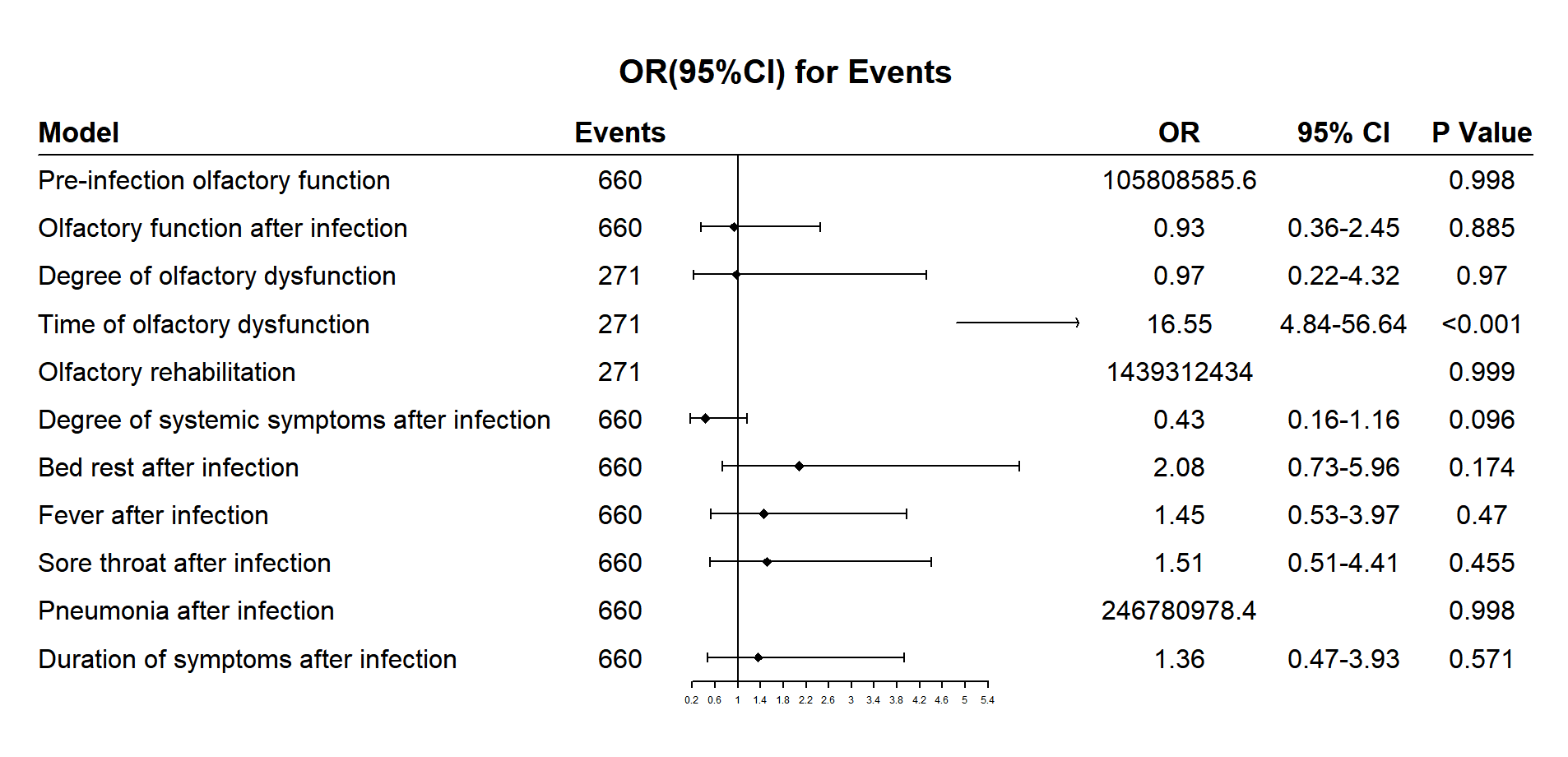
***

***Figure S3. Forest plot of clinical symptoms and vaccination status before and after COVID-19 infection***. Models adjusted for age, gender, allergic rhinitis, whether or not vaccinated, and pre-infection olfactory function; OR, Odds Ratio; CI, confidence intervals.


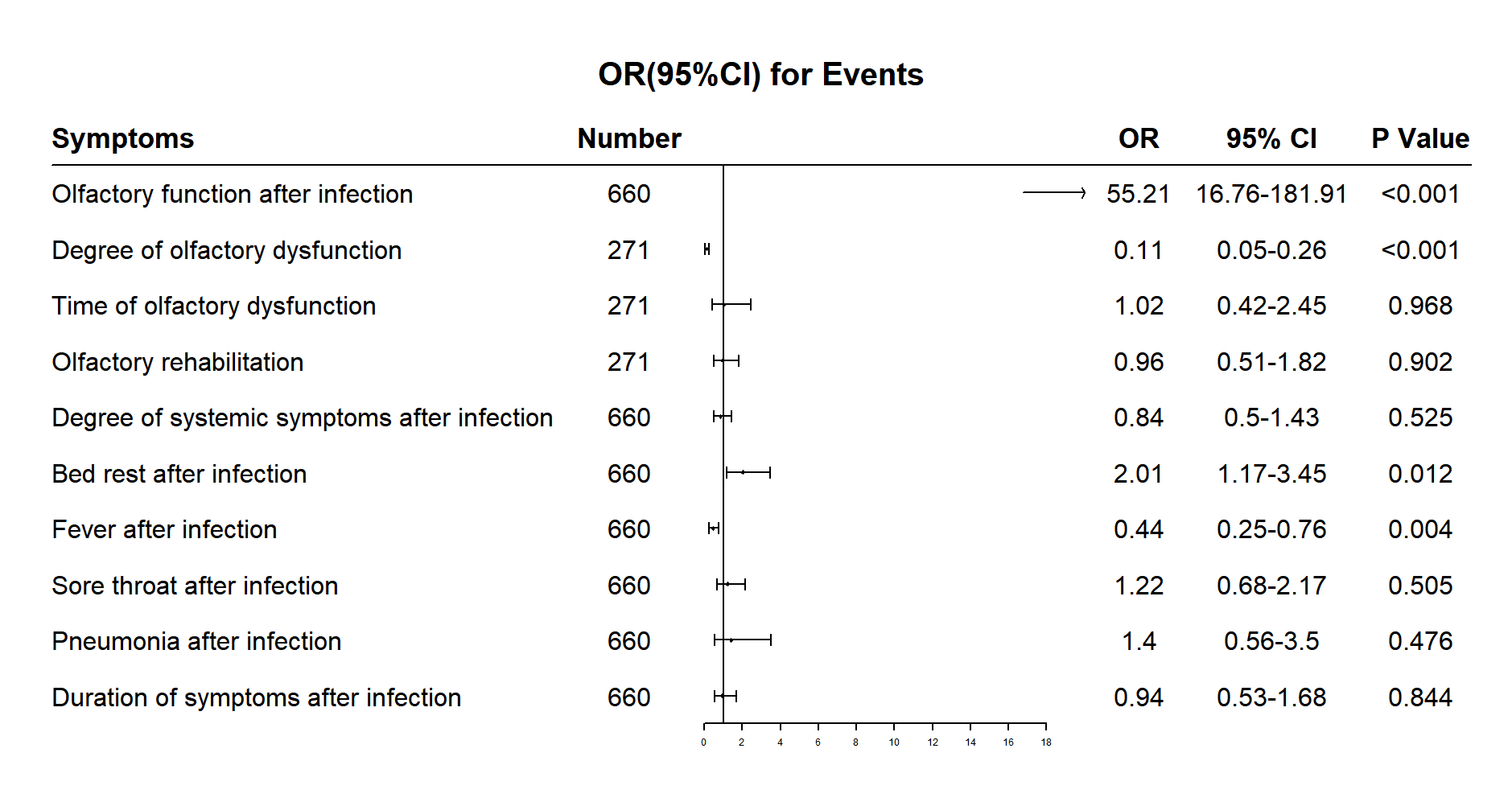


***Figure S4. Forest plot of clinical symptoms and olfactory after COVID-19 infection.*** Models adjusted for age, gender, allergic rhinitis, whether or not vaccinated, and pre-infection olfactory function; OR, Odds Ratio; CI, confidence intervals.

STROBE Statement—Checklist of items that should be included in reports of ***cohort studies***

|  | Item No | Recommendation | Page No |
| --- | --- | --- | --- |
| **Title and abstract** | 1 | (*a*) Indicate the study’s design with a commonly used term in the title or the abstract | 1 |
|  |  | (*b*) Provide in the abstract an informative and balanced summary of what was done and what was found | 2 |
| Introduction | | | |
| Background/rationale | 2 | Explain the scientific background and rationale for the investigation being reported | 3 |
| Objectives | 3 | State specific objectives, including any prespecified hypotheses | 4 |
| Methods | | | |
| Study design | 4 | Present key elements of study design early in the paper | 4 |
| Setting | 5 | Describe the setting, locations, and relevant dates, including periods of recruitment, exposure, follow-up, and data collection | 4 |
| Participants | 6 | (*a*) Give the eligibility criteria, and the sources and methods of selection of participants. Describe methods of follow-up | ~~4~~ |
|  |  | (*b*) For matched studies, give matching criteria and number of exposed and unexposed | NA |
| Variables | 7 | Clearly define all outcomes, exposures, predictors, potential confounders, and effect modifiers. Give diagnostic criteria, if applicable | 4 |
| Data sources/ measurement | 8* | For each variable of interest, give sources of data and details of methods of assessment (measurement). Describe comparability of assessment methods if there is more than one group | 4,5 |
| Bias | 9 | Describe any efforts to address potential sources of bias | 4,5 |
| Study size | 10 | Explain how the study size was arrived at | ~~4~~ |
| Quantitative variables | 11 | Explain how quantitative variables were handled in the analyses. If applicable, describe which groupings were chosen and why | 4,5 |
| Statistical methods | 12 | (*a*) Describe all statistical methods, including those used to control for confounding | 4,5 |
|  |  | (*b*) Describe any methods used to examine subgroups and interactions | 4,5 |
|  |  | (*c*) Explain how missing data were addressed | NA |
|  |  | (*d*) If applicable, explain how loss to follow-up was addressed | NA |
|  |  | (*e*) Describe any sensitivity analyses | NA |
| Results | | |  |
| Participants | 13* | (a) Report numbers of individuals at each stage of study—eg numbers potentially eligible, examined for eligibility, confirmed eligible, included in the study, completing follow-up, and analysed | 5,6 |
|  |  | (b) Give reasons for non-participation at each stage | NA |
|  |  | (c) Consider use of a flow diagram | NA |
| Descriptive data | 14* | (a) Give characteristics of study participants (eg demographic, clinical, social) and information on exposures and potential confounders | 5,6 |
|  |  | (b) Indicate number of participants with missing data for each variable of interest | NA |
|  |  | (c) Summarise follow-up time (eg, average and total amount) | NA |
| Outcome data | 15* | Report numbers of outcome events or summary measures over time | 5 |

| Main results | 16 | (*a*) Give unadjusted estimates and, if applicable, confounder-adjusted estimates and their precision (eg, 95% confidence interval). Make clear which confounders were adjusted for and why they were included | 7,10 |
| --- | --- | --- | --- |
|  |  | (*b*) Report category boundaries when continuous variables were categorized | NA |
|  |  | (*c*) If relevant, consider translating estimates of relative risk into absolute risk for a meaningful time period |  |
| Other analyses | 17 | Report other analyses done—eg analyses of subgroups and interactions, and sensitivity analyses | 13 |
| Discussion | | | |
| Key results | 18 | Summarise key results with reference to study objectives | 13 |
| Limitations | 19 | Discuss limitations of the study, taking into account sources of potential bias or imprecision. Discuss both direction and magnitude of any potential bias | 13 |
| Interpretation | 20 | Give a cautious overall interpretation of results considering objectives, limitations, multiplicity of analyses, results from similar studies, and other relevant evidence | 12 |
| Generalisability | 21 | Discuss the generalisability (external validity) of the study results | 11,12 |
| Other information | | | |
| Funding | 22 | Give the source of funding and the role of the funders for the present study and, if applicable, for the original study on which the present article is based | 14 |

*Give information separately for exposed and unexposed groups.

**Note:** An Explanation and Elaboration article discusses each checklist item and gives methodological background and published examples of transparent reporting. The STROBE checklist is best used in conjunction with this article (freely available on the Web sites of PLoS Medicine at http://www.plosmedicine.org/, Annals of Internal Medicine at http://www.annals.org/, and Epidemiology at http://www.epidem.com/). Information on the STROBE Initiative is available at http://www.strobe-statement.org.

Participant Flow Diagram:


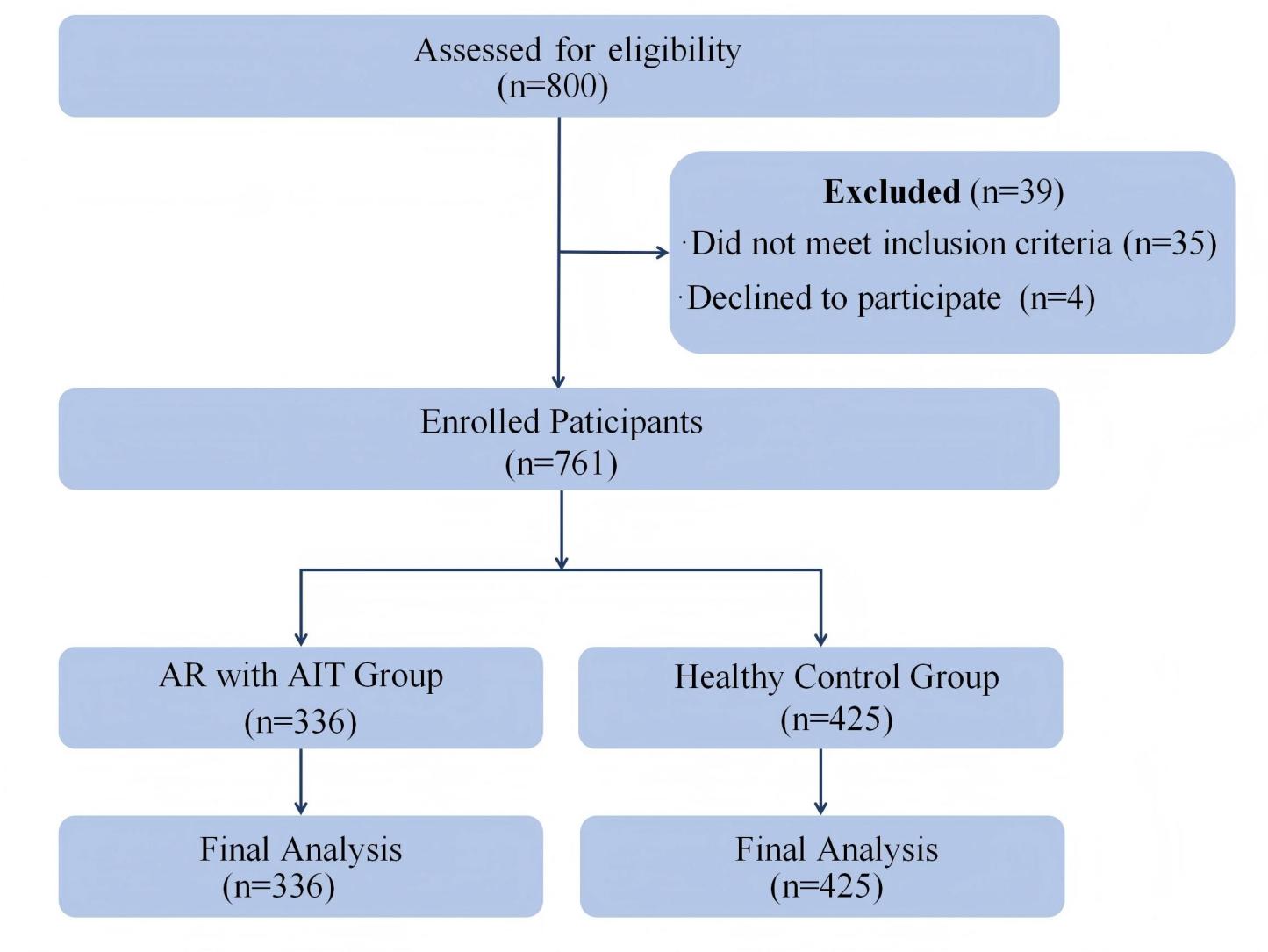


**COVID-19 Quality of Life Assessment Scale.**

1. Are you in the COVID-19 period or have you recovered.

in the COVID-19/recovered.

1. In the following activities, choose 3. In the past week, has COVID-19 influenced you to the greatest extent?

| Cycling | Playing table tennis | Eating |
| --- | --- | --- |
| Reading | Playing with pets | Use a vacuum cleaner |
| Shopping | Playing with children/grandchildren | Visit friends or relatives |
| Do household maintenance | Participating in group sports | Take a walk outside |
| Do household chores | Driving | Taking children to school |
| Enter/exit air-conditioned rooms | Singing | Outdoor activities |
| Watching TV | Engaging in social activities | Work |
| Exercise or Sports | Sexual activity | Sitting outdoors |
| Morning Exercise | Badminton | Taking children to the park |
| Using a computer | Chat | Being in smoking environment |

3.In the past week, to what extent were the three activities selected above plagued by COVID-19? Please adjust the severity of symptoms from 0 no distress to 10 complete distress

Activity 1:

Activity 2:

Activity 3:

1. To what extent are you troubled by the following issues?According to the severity of symptoms, 0 is no trouble and 10 is completely trouble.

| Sleep quality | According to the severity of symptoms, 0 is no trouble and 10 is completely trouble. |
| --- | --- |
| Difficulty falling asleep: |  |
| Wake up at night |  |
| Poor sleep quality at night |  |
| emotional issues | According to the severity of symptoms, 0 is no trouble and 10 is completely trouble. |
| Irritable |  |
| Depressed |  |
| Impatience or unease in the heart |  |
| Feeling embarrassed due to symptoms |  |
| Other Symptoms | According to the severity of symptoms, 0 is no trouble and 10 is completely trouble. |
| Be deficient in energy |  |
| Tired |  |
| Decreased work ability |  |
| Difficulty concentrating |  |
| Headache |  |
